# Analysis of breast region segmentation in thermal images using U-Net deep neural network variants

**DOI:** 10.3389/fbinf.2025.1609004

**Published:** 2025-10-10

**Authors:** Rafhanah Shazwani Rosli, Mohamed Hadi Habaebi, Md Rafiqul Islam, Mohammed Abdulla Salim Al Hussaini

**Affiliations:** 1 IoT and Wireless Communication Protocols Laboratory, Department of Electrical and Computer Engineering, International Islamic University Malaysia (IIUM), Kuala Lumpur, Malaysia; 2 Faculty of Computer Studies, Arab Open University (AOU), Muscat, Oman

**Keywords:** breast region segmentation, thermal images, thermography, deep learning, deep neural network, artificial intelligence, U-Net, U-Net with spatial attention

## Abstract

**Introduction:**

Breast cancer detection using thermal imaging relies on accurate segmentation of the breast region from adjacent body areas. Reliable segmentation is essential to improve the effectiveness of computer-aided diagnosis systems.

**Methods:**

This study evaluated three segmentation models—U-Net, U-Net with Spatial Attention, and U-Net++—using five optimization algorithms (ADAM, NADAM, RMSPROP, SGDM, and ADADELTA). Performance was assessed through k-fold cross-validation with metrics including Intersection over Union (IoU), Dice coefficient, precision, recall, sensitivity, specificity, pixel accuracy, ROC-AUC, PR-AUC, and Grad-CAM heatmaps for qualitative analysis.

**Results:**

The ADAM optimizer consistently outperformed the others, yielding superior accuracy and reduced loss. Among the models, the baseline U-Net, despite being less complex, demonstrated the most effective performance, with precision of 0.9721, recall of 0.9559, specificity of 0.9801, ROC-AUC of 0.9680, and PR-AUC of 0.9472. U-Net also achieved higher robustness in breast region overlap and noise handling compared to its more complex variants. The findings indicate that greater architectural complexity does not necessarily lead to improved outcomes.

**Discussion:**

This research highlights that the original U-Net, when trained with the ADAM optimizer, remains highly effective for breast region segmentation in thermal images. The insights contribute to guiding the selection of suitable deep learning models and optimizers for medical image analysis, with the potential to enhance the efficiency and accuracy of breast cancer diagnosis using thermal imaging.

## 1 Introduction

Breast cancer remains a global health concern, underscoring the critical importance of early detection for improved patient prognosis (Sung et al., 2021). Recent advancements in medical imaging, particularly thermal imaging, offer potential for enhancing early detection capabilities (Allugunti, 2022). However, the effectiveness of these technologies relies heavily on the precision of image segmentation, particularly in isolating the breast region from surrounding anatomical structures (Dafni Rose et al., 2022). This study addresses the pressing need for accurate and efficient breast region segmentation in thermal images, with the overarching goal of advancing early breast cancer detection.

The motivation for this research stems from the recognition that thermal imaging holds promise in detecting breast cancer early, and its success hinges on the precision of the segmentation process. To optimize thermal imaging pre-processing, we focus on leveraging advanced deep learning techniques, specifically U-Net variants. U-Net’s symmetrical expansive pathway proves advantageous, enabling precise delineation of intricate boundaries, a crucial requirement in medical imaging (Zhou et al., 2018). The decision to employ U-Net variants is informed by their efficiency, precision, and adaptability, especially in the challenging task of segmenting the breast region in thermal images.

In contrast to alternative models like SegNet, DeepLabv3+, Mask R-CNN, and EfficientNet, U-Net variants demonstrate superior efficiency and adaptability for sparse data, making them a preferred choice for this study (Badrinarayanan et al., 2017). DeepLabv3+ and Mask R-CNN, while powerful, pose challenges such as larger training datasets and substantial computational loads, limiting their suitability for our specific application (Chen et al., 2018; He et al., 2017). The adoption of U-Net variants is poised to significantly enhance the accuracy and efficiency of breast region segmentation, aligning with the objectives of this research (Tan and Le, 2019).

Breast region segmentation in thermal images involves distinguishing the breast area from surrounding body parts, a complex task given variations in size, shape, and orientation across individuals (Soomro et al., 2022). Several deep learning models, including U-Net, U-Net with Spatial Attention, and U-Net++ (Nested U-Net), have shown promise in image segmentation but have not been thoroughly explored for breast region segmentation in thermal images (Azad et al., 2022; Radhi and Kamil, 2023; Punn and Agarwal, 2022; Liu et al., 2022; Gu et al., 2022; Islam Sumon et al., 2023; Yin et al., 2022; Micallef et al., 2021; Mokhtar et al., 2023; Gargari et al., 2022; Zhao et al., 2022). This study not only evaluates the performance of these models but also conducts a comprehensive comparison of different optimization algorithms, recognizing the optimizer’s pivotal role in training deep learning models.

By systematically evaluating various optimizers and identifying the most effective one for training segmentation models, this study aims to provide a holistic assessment of the segmentation task. The research presents a comprehensive evaluation of U-Net, U-Net with Spatial Attention, and U-Net++ for breast region segmentation in thermal images, coupled with a thorough comparison of different optimizers. The insights generated from this study are poised to contribute significantly to the advancement of early breast cancer detection technologies, benefiting researchers and practitioners in the fields of medical diagnostics and artificial intelligence.

## 2 Related work

Breast region segmentation in thermal images has emerged as a pivotal area of research, given its potential in breast cancer detection. Diverse methodologies, ranging from conventional image processing techniques to cutting-edge deep learning models, have been proposed to improve the precision and efficiency of segmentation. The significance of deep learning methodologies, particularly their potential to bring beneficial effects in enhancing computer-aided medical diagnosis, is emphasized in (Al Husaini et al., 2023).

In a study employing Distance-based Metrics and High-Temperature Region-based Adaptive Thresholding (DM-HTRAT) (Venkatachalam et al., 2023), an accuracy of 96.5% in breast boundary segmentation was achieved, contributing to more reliable and effective detection of breast abnormalities. However, limitations include susceptibility to unclear boundaries, a low signal-to-noise ratio, and poor contrast in thermal images.

Another study proposed an automatic segmentation algorithm (Adel et al., 2018) that successfully segmented all types of breasts with an accuracy of 98.73%. While demonstrating faster runtimes than the Hough transform, challenges may arise in real-time applications requiring instantaneous results.

A comprehensive review of various image processing techniques for automatic segmentation of clinically significant Regions of Interest (ROIs) emphasized the importance of automated segmentation for fast and reproducible analysis (Singh and Arora, 2020). The review also highlighted the potential of deep learning for effective computer-aided medical diagnosis, acknowledging the limitations of human-based diagnoses influenced by factors such as narcissus effect, negligence, visual exhaustion, and mental workload.

A proposed methodology relying on local analysis to mitigate the impact of global noise achieved a new alternative for automatic segmentation of thermal breast images with 77.3% accuracy (Sánchez-Ruiz et al., 2018). However, errors were observed in images with low contrast in the breast region and those depicting amorphous breast structures.

Autoencoder-like convolutional and deconvolutional neural networks (C-DCNN) demonstrated the capability to learn essential features of breast regions and delineate them in thermal images (Guan et al., 2018). The study suggested a need for an improved evaluation metric to effectively assess the quality of the breast segmentation model.

The MultiResUnet deep-learning segmentation model exhibited an average accuracy of 91.47%, surpassing the autoencoder by about 2% (Lou et al., 2019). However, limitations in small breast segmentation, IoU errors, data augmentation, and manual challenges were identified, suggesting areas for improvement.

Utilizing Genetic Algorithms (GA) with a fitness function based on cardioids, a method successfully separated the breast region in 52 out of 58 images without manual seed point selection (Mendes et al., 2020). However, challenges were faced with ellipse techniques and metallic markers, and the algorithm required 60 s for optimal results.

U-Net Convolutional Neural Networks demonstrated efficiency for Region of Interest (ROI) segmentation, achieving an accuracy of 98.24% over frontal views and 93.6% over lateral views (Carlos de Carvalho et al., 2023). Notably, the efficacy of the method decreased when applied to lateral views.

A study incorporating Vector Pooling Block (VPB) and AVG-MAX VPB in Convolutional Neural Networks (U-Net, AlexNet, ResNet18, GoogleNet) achieved impressive results, including a global accuracy of 99.2% (Mohamed et al., 2022). However, the study noted the need for more efficient exploration of the pooling layer’s effect in Convolutional Neural Networks (CNNs) within the existing literature.

## 3 Materials and methods

This section details the methodological framework adopted in this study, encompassing the dataset acquisition and preparation, model architectures, experimental setup, and the subsequent training and evaluation processes.

### 3.1 Dataset acquisition and preparation

The DMR-IR database is a publicly accessible repository containing multimodal breast examination data, including infrared thermography, digital mammography, and clinical records. For this study, only the frontal thermal images of 130 patients were used, acquired under the First Static Protocol to ensure standardized conditions. Patients included both healthy controls and individuals with benign and malignant breast lesions, thereby introducing variability essential for robust model evaluation. The infrared images were captured using a FLIR SC620 camera, with a sensitivity of <0.04 °C and a temperature range of 40 °C–500 °C, at a resolution of 640 × 480 pixels. To minimize external variability, all acquisitions followed a controlled clinical protocol, where patients were acclimatized in a room maintained at 20 °C–23 °C for 15 min before imaging. Manual annotations were performed by the study authors to generate ground-truth masks, with cross-verification among annotators to reduce bias. While not performed by certified radiologists, this procedure was explicitly designed for experimental, non-clinical purposes. The use of the DMR-IR database ensures transparency and reproducibility, as the dataset is publicly available online (http://visual.ic.uff.br/dmi), allowing independent research.

In this study, the dataset utilized for experimentation was obtained from an accessible online database. The main objective of this research revolves around the comparative analysis of three U-Net deep neural network variants. Therefore, leveraging a pre-existing dataset rather than dedicating resources to the creation of a new one is a strategic decision which allowed this study to focus on the main objective.

#### 3.1.1 Data acquisition

A collection of thermal breast images from 130 patients was acquired from the Database for Breast Research with Infrared Image (DMR-IR) as presented by (Silv et al., 2014). Specifically, frontal breast thermal images captured under the First Static Protocol. The DMI-IR incorporates infrared images, digitalized mammograms, and clinical data acquired from Antônio Pedro University Hospital patients. These patients come from the screening department and the gynecology department. The DMI-IR contains data on healthy patients as well as patients with breast diseases, including cancer.

The infrared images, henceforth referred to as ‘thermal images’ were obtained by a FLIR thermal camera, model SC620, with a sensitivity of less than 0.04 °C and a capture standard of 40 °C–500 °C. The pixel dimensions of the infrared images are 640 × 480. The procurement of the images and their use in research have been approved by the hospital’s Ethical Committee and registered with the Brazilian Ministry of Health under the following CAAE number: 01042812.0.0000.5243. The DMR-IR is accessible via an online user-friendly interface (http://visual.ic.uff.br/dmi) for managing and retrieving data from breast examinations and clinical data from voluntary patients.

#### 3.1.2 Annotation and mask generation

**FIGURE 1:**
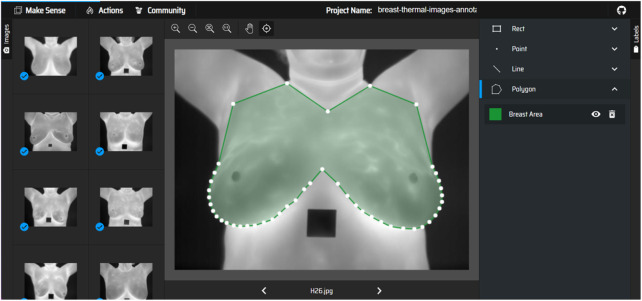
Annotation of breast area on breast thermal images.

Manual annotations were performed by the authors to prepare masks for thermal images. The breast region in each image was delineated, creating a mask that highlights the breast area and excludes other regions like the armpit, neck, and lower chest. The masks served as the ground truth for training the segmentation models. It is important to highlight that the annotation was performed only for the purpose of experimentation, and not for the use in clinical setup, as it was not performed by a certified technician. Figure 1 shows the screenshot of annotation of breast area from breast thermal image.

**FIGURE 2:**
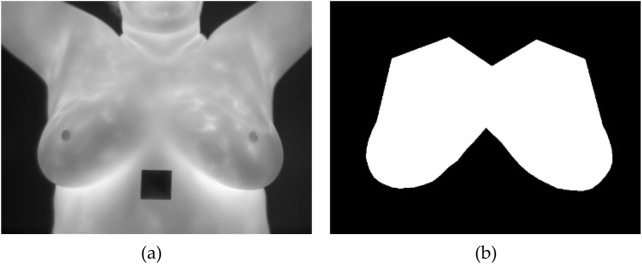
**(a)** Breast thermal image; **(b)** Corresponding binary mask.

Once the annotations are completed, they are exported in a VGG JSON format, which is a structured format to represent these annotations. After obtaining the annotations in the VGG JSON format, it is then used to generate binary masks for each annotated image. A binary mask is a black and white representation where the regions of interest are shown in white, and everything else is black. A sample of an unsegmented breast thermal image and its corresponding binary mask is shown in Figure 2.

#### 3.1.3 Data preprocessing

Data preprocessing was conducted on each thermal image and its corresponding binary mask prior to the segmentation process. The process involved adjusting the size of the images to a consistent dimension of 256x256 pixels, standardizing the pixel values to fall within the range of 0–1, and producing diverse versions of data augmentation from both the images and masks. The inclusion of this data augmentation step enhances the model’s ability to generalize by introducing greater diversity into the training dataset.

The following transformations were conducted on both the images and their associated binary masks.

a. Rotation: To accommodate diverse breast orientations, images undergo random rotations of up to 20°.
b. Width and Height Shifts: Images are shifted both horizontally and vertically by a maximum of 10% of their respective dimensions, aiding the model in identifying off-center region.
c. Shear Transformation: The images are slanted with an intensity of up to 0.2, introducing a skewing effect.
d. Zooming: Random zooming in or out of images by a factor of up to 20%, helping the model adapt to breast region of different scales.
e. Flipping: Images are flipped both horizontally and vertically, useful for datasets where breast orientation is not consistent.
f. Pixel Fill Strategy: After transformations like rotation or shifts, new pixels were created. The ‘reflect’ strategy is used to mirror the edge pixels of the image.

### 3.2 Model architectures

To improve breast region segmentation in thermal images, this study evaluated three distinct deep learning architectures. Each of these models is recognized for their image segmentation ability.

#### 3.2.1 U-net

**FIGURE 3:**
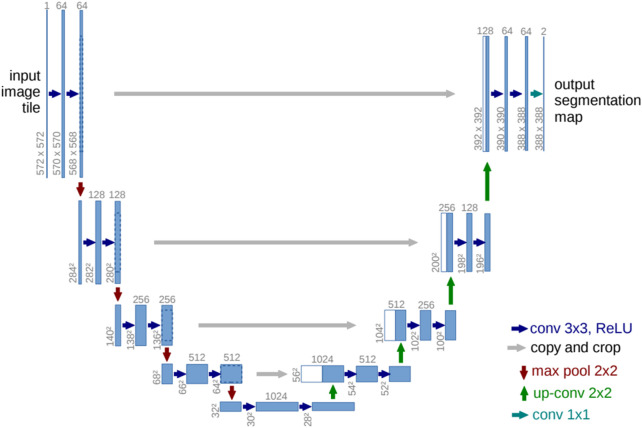
U-net model (Ronneberger et al., 2015).

U-Net, as introduced in paper (Ronneberger et al., 2015), is a deep learning architecture specifically designed for biomedical image segmentation. To address the challenge of effectively training deep neural networks with a limited number of annotated samples, the authors proposed a data augmentation-based approach. U-Net’s architecture includes a contracting path for context assimilation and an expanding path for granular localization. Despite training on a limited image dataset, U-Net outperformed previous methods. The network’s structure consists of 23 convolutional layers. The U-Net model for the base resolution of 32x32 pixels is depicted in Figure 3. Each blue rectangle in this diagram represents a multichannel feature map, with the channel count indicated atop each rectangle and the x-y dimension indicated at its lower left. The duplicated feature maps depicted in white are denoted by arrows, which represent various operations.

#### 3.2.2 U-net with spatial attention

**FIGURE 4:**
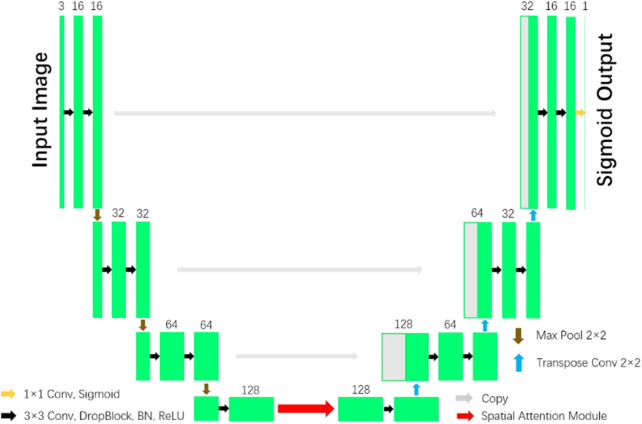
U-Net with Spatial Attention model (Guo et al., 2021).

In the paper (Guo et al., 2021), a network with reduced computational complexity known as Spatial Attention U-Net (SA-UNet) has been introduced. This network does not require a large number of annotated training samples. Alternatively, it can be utilized in a data augmentation methodology to optimize the utilization of the existing annotated samples. One notable characteristic of SA-UNet is its integration of a spatial attention module. The attention map along the spatial dimension is inferred by this module, then multiplied with the input feature map to enable adaptive feature refinement. Furthermore, to mitigate the issue of overfitting, the neural network utilizes structured dropout convolutional blocks as a substitute for the original convolutional blocks found in the U-Net architecture. Figure 4 illustrates the SA-UNet model, which consists of a U-shaped encoder on the left side and a decoder on the right side.

Each stage of the encoder consists of a structured dropout convolutional block and a 2 × 2 max pooling operation. In each convolutional block, the convolutional layer is succeeded by a DropBlock, a batch normalization (BN) layer, and a rectified linear unit (ReLU). Subsequently, the max pooling operation is employed to down-sample the data with a stride size of 2. In each down-sampling step, the number of feature channels is doubled. Each step in the decoder involves a 2 × 2 transposed convolution operation for up-sampling and reduces the number of feature channels by half. This is followed by concatenation with the corresponding feature map from the encoder, and then a structured dropout convolutional block is applied. The inclusion of a spatial attention module is implemented in the intermediate stage between the encoder and the decoder. In the ultimate layer, the utilization of a 1x1 convolution and the application of the Sigmoid activation function are employed to obtain the resulting segmentation map.

#### 3.2.3 U-Net++

**FIGURE 5:**
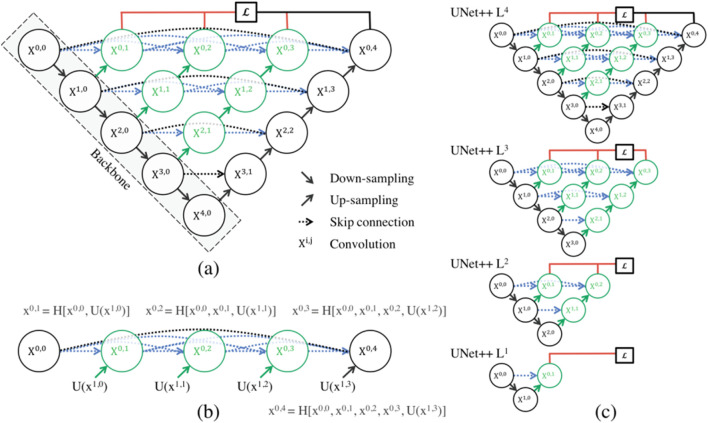
**(a)** UNet++ is a neural network that bridges the semantic gap between encoder and decoder feature maps before fusion. It uses nested dense convolutional blocks to bridge the gap between (X0,0, X1,3). The graphical abstract shows black for original U-Net, green and blue for skip pathways, and red for deep supervision. The components distinguish UNet++ from U-Net. **(b)** Detailed analysis of the first UNet++ skip pathway. **(c)** UNet++ can be pruned at the time of inference if it is trained under intensive supervision (Zhou et al., 2018).

The paper (Zhou et al., 2018) introduces UNet++, a powerful medical image segmentation architecture with a deeply-supervised encoder-decoder network. The architecture connects encoder and decoder sub-networks through nested, dense skip pathways, aiming to reduce the semantic gap between feature maps. The optimizer handles easier learning tasks when feature maps from decoder and encoder networks are semantically similar. Figure 5a depicts an overview of the proposed architecture. As can be seen, UNet++ begins with an encoder sub-network or backbone, which is followed by a decoder sub-network. What differentiates UNet++ from U-Net (the black components in Figure 5a) are the redesigned skip pathways that connect the two sub-networks (shown in green and blue in Figure 5b) and the use of deep supervision (shown in red in Figure 5c).

### 3.3 Experimental setup

#### 3.3.1 Software configuration

The experimental setup utilized advanced software tools and frameworks to conduct the research. The primary software components included:

a. Operating System: The experiments were conducted on a system running the latest version of Windows 11, providing a stable and user-friendly environment for the research tasks.
b. Deep Learning Frameworks: State-of-the-art deep learning frameworks such as TensorFlow and Keras were employed for model development, training, and evaluation. These frameworks offered a rich set of functionalities, making it possible to implement complex neural network architectures and algorithms efficiently.
c. Image Processing Libraries: OpenCV, a powerful open-source computer vision library, was employed for various image processing tasks. It provided essential tools for image manipulation, feature extraction, and visualization, crucial for preprocessing thermal images and analyzing the results.
d. Data Management: Python libraries like NumPy and Pandas were utilized for efficient data manipulation and analysis. NumPy facilitated numerical operations, while Pandas allowed structured data handling, enabling seamless organization and processing of experimental data.
e. Visualization: Matplotlib, a versatile plotting library in Python, was used for generating visualizations such as graphs, charts, and figures. It played a vital role in presenting experimental results and analyzing trends in the data.

#### 3.3.2 Hardware configuration

The experimental setup was supported by robust hardware configurations, ensuring efficient computation and data processing. The key components of the hardware setup included:

a. Processor: An Intel 13th Gen Core i9-13900HX processor with a base clock speed of 2.20 GHz provides substantial computing power. Its high processing capabilities enabled swift execution of complex algorithms and simulations.
b. Memory: The system was equipped with 32 GB of RAM, allowing for the seamless handling of large datasets and resource-intensive deep learning tasks. The ample memory capacity facilitated smooth multitasking and efficient training of neural networks.
c. Graphics Processing Unit (GPU): The experimental setup featured an NVIDIA GeForce RTX 4080 Laptop GPU. This high-performance GPU accelerated deep learning computations, enabling the training of complex neural networks and the execution of computationally intensive tasks.

### 3.4 Model training and evaluation

**FIGURE 6:**
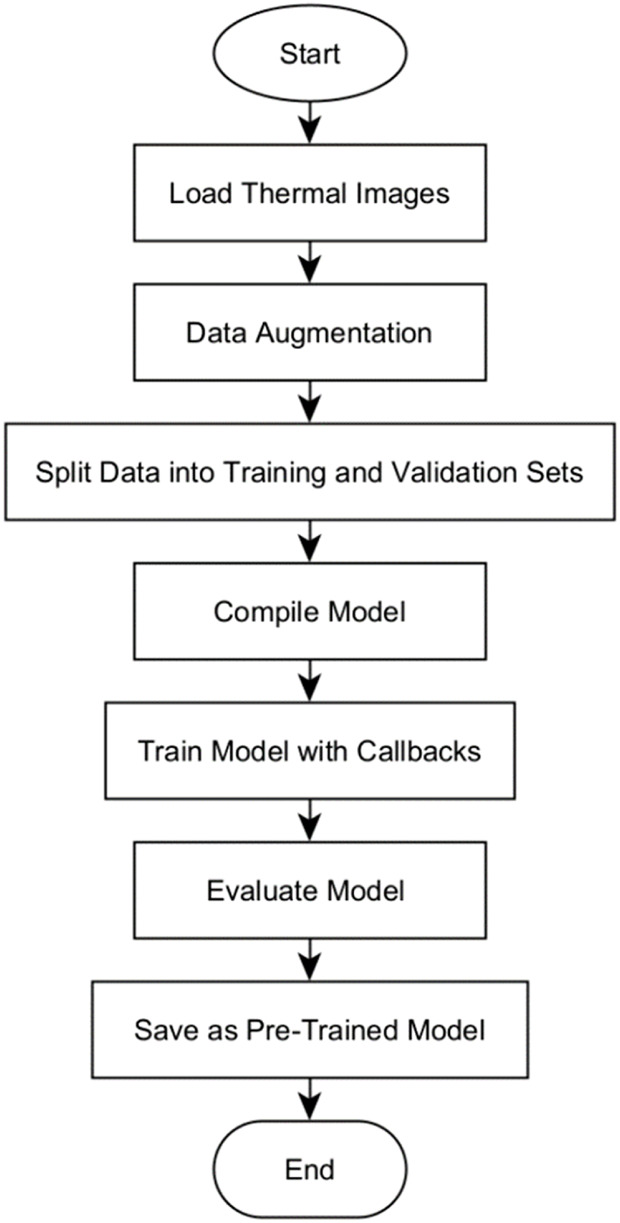
Flowchart for the model’s training and evaluation algorithm.

The training and evaluation of the U-Net, U-Net with Spatial Attention, and U-Net++ models were conducted based on the flowchart of the algorithm as illustrated in Figure 6.

The algorithm begins by loading grayscale images and its corresponding true masks from specified directories, resizes, and normalizes their pixel values. To augment the dataset, the images and masks are subjected to various transformations as explained in Section 4.1.3, thereby introducing variability into the training data. A stratified split approach was adopted to split the data into training and validation sets, with 20% of the augmented dataset reserved for validation. This is specifically for experimental comparison between models. This allocation, while not subjected to active experimentation, was designed to ensure a balanced representation of diverse classes in both training and validation sets. The model is then initialized based on the specific model type (U-Net, U-Net with Spatial Attention, or U-Net++). The model is compiled using the binary cross-entropy loss function and accuracy metric, crucial for measuring segmentation precision.

Five different optimizers were evaluated comparatively: ADAM, NADAM, RMSPROP, SGDM, and ADADELTA. The evaluation of the specific optimizers was based on their widespread usage and documented effectiveness in various deep learning applications, particularly in image segmentation tasks. The mathematical equations that describe how the optimizers update the model weights during training are as follows.

#### 3.4.1 ADAM (adaptive moment estimation)

ADAM combines the advantages of both momentum-based optimization and RMSProp. It maintains adaptive learning rates for each parameter and keeps an exponentially decaying average of past gradients. The Equations 1–5 for ADAM, as described by (Kingma and Ba, 2014), are as follows:

$$m_{t}=\beta_{1}\cdot m_{t-1}+\left(1-\beta_{1}\right)\cdot g_{t}$$ (1)

$$v_{t}=\beta_{2}\cdot v_{t-1}+\left(1-\beta_{2}\right)\cdot g_{t}^{2}$$ (2)

$$m_{t}^{corrected}=\frac{m_{t}}{1-\beta_{1}^{t}}$$ (3)

$$v_{t}^{corrected}=\frac{v_{t}}{1-\beta_{2}^{t}}$$ (4)

$$\theta_{t}=\theta_{t-1}-\frac{\alpha \cdot m_{t}^{corrected}}{\sqrt{v_{t}^{corrected}}+\epsilon }$$ (5)

Where:

$m_{t}$ and $v_{t}$ are the first and second moments estimates,

$g_{t}$ is the gradient at time step t,

$\beta_{1}$ and $\beta_{2}$ are exponential decay rates for the moment estimates,

α is the learning rate,

ϵ is a small constant added to prevent division by zero.

#### 3.4.2 NADAM (nesterov-adam)

NADAM optimizer combines Nesterov’s accelerated gradient with the benefits of ADAM. It uses the same equations as ADAM but with Nesterov’s momentum applied to the gradients before calculating $m_{t}$ . In standard momentum, the update rule for a parameter θ is given by Equations 6, 7:

$$v_{t}=\beta \cdot v_{t-1}+\alpha \cdot \nabla f\left(\theta_{t-1}+\beta \cdot v_{t-1}\right)$$ (6)

$$\theta_{t}=\theta_{t-1}-v_{t}$$ (7)

Where:

α is the learning rate,

$\nabla f\left(\theta_{t-1}+\beta \cdot v_{t-1}\right)$ is the gradient of the objective function at the predicted future position, 

β is the momentum parameter.

Nesterov momentum modifies this approach by calculating the gradient at a “lookahead” position (Dozat, 2016) as given by Equations 8–10.

$$\theta_{\text{lookahead}}=\theta_{t-1}+\beta \cdot v_{t-1}$$ (8)

$$v_{t}=\beta \cdot v_{t-1}+\alpha \cdot \nabla f\left(\theta_{\text{lookahead}}\right)$$ (9)

$$\theta_{t}=\theta_{t-1}-v_{t}$$ (10)

#### 3.4.3 RMSPROP (root mean square propagation)

RMSPROP adapts the learning rates for each parameter based on the average of recent magnitudes of the gradients. It prevents vanishing or exploding gradients by scaling the gradients with a moving average of their squared values, as captured by Equations 11, 12 (Tieleman and Hinton, 2012).

$$E{\left[g^{2}\right]}_{t}=\beta \cdot E{\left[g^{2}\right]}_{t-1}+\left(1-\beta \right)\cdot g_{t}^{2}$$ (11)

$$\theta_{t}=\theta_{t-1}-\frac{\alpha \cdot g_{t}}{\sqrt{E{\left[g^{2}\right]}_{t}+\epsilon }}$$ (12)

Where:

$E{\left[g^{2}\right]}_{t}$ is the moving average of squared gradients, 

β is the decay rate for the moving average, 

α is the learning rate, 

ϵ is a small constant added to prevent division by zero.

#### 3.4.4 SGDM (stochastic gradient descent with momentum)

SGDM incorporates momentum, allowing the optimizer to accumulate velocity and dampens oscillations. The momentum term helps the optimizer traverse through local minima more effectively. The SGDM Equations 13, 14 are derived based on the concept of accumulated gradients (Qian, 1999).

$$v_{t}=\beta \cdot v_{t-1}+\alpha \cdot g_{t}$$ (13)

$$\theta_{t}=\theta_{t-1}-v_{t}$$ (14)

Where:

$v_{t}$ is the velocity or momentum term, 

β is the momentum coefficient, 

α is the learning rate, 

$g_{t}$ is the gradient at time step t.

#### 3.4.5 ADADELTA

ADADELTA dynamically adapts the learning rates based on past gradients without the need for manual tuning. It utilizes moving averages of both squared gradients and parameter updates to scale the gradients effectively, as shown in Equations 15–17 (Zeiler, 2012).

$$E{\left[g^{2}\right]}_{t}=\rho \cdot E{\left[g^{2}\right]}_{t-1}+\left(1-\rho \right)\cdot g_{t}^{2}$$ (15)

$$\Delta \theta_{t}=-\frac{\sqrt{\Delta \theta_{t-1}^{2}+\epsilon }}{\sqrt{E{\left[g^{2}\right]}_{t}+\epsilon }}\cdot g_{t}$$ (16)

$$\theta_{t}=\theta_{t-1}+\Delta \theta_{t}$$ (17)

Where:

$E{\left[g^{2}\right]}_{t}$ is the exponentially decaying average of squared gradients,

ρ is the decay rate,

$\Delta \theta_{t}$ is the parameter update.

All three models were trained using each of the five optimizers. The training was carried out in a controlled environment, ensuring the same number of epochs, batch size, and data augmentation techniques. The model is trained using the training data for a total of 30 epochs and a batch size of 20. The choice of 30 epochs was based on preliminary experiments, where we observed that all three models consistently converged within this range without signs of overfitting. Using a fixed number of epochs ensured fairness and comparability across models and optimizers. Moreover, callbacks were implemented to dynamically adjust the learning rate during training. While the dataset size (130 patients) is relatively small, it was chosen due to its availability in the DMR-IR database and the variability it provides across healthy, benign, and malignant cases. This limitation is acknowledged, but the use of data augmentation and k-fold cross-validation helped to mitigate its impact. The number of epochs and batch size, while not subjected to active experimentation, were chosen specifically to facilitate a fair and systematic experimental comparison between the models. Callbacks function, are used to dynamically adjust the learning rate during training based on the validation loss, allowing the model to adapt as it learns. The start and end times of the training are recorded, and the total training time is computed to assess the computational efficiency of the training process. Upon completion of the training, the performance of the model is evaluated on the entire dataset, and the final loss and accuracy are observed. The results are presented in Section 5.1.

### 3.5 Quantitative analysis

**FIGURE 7:**
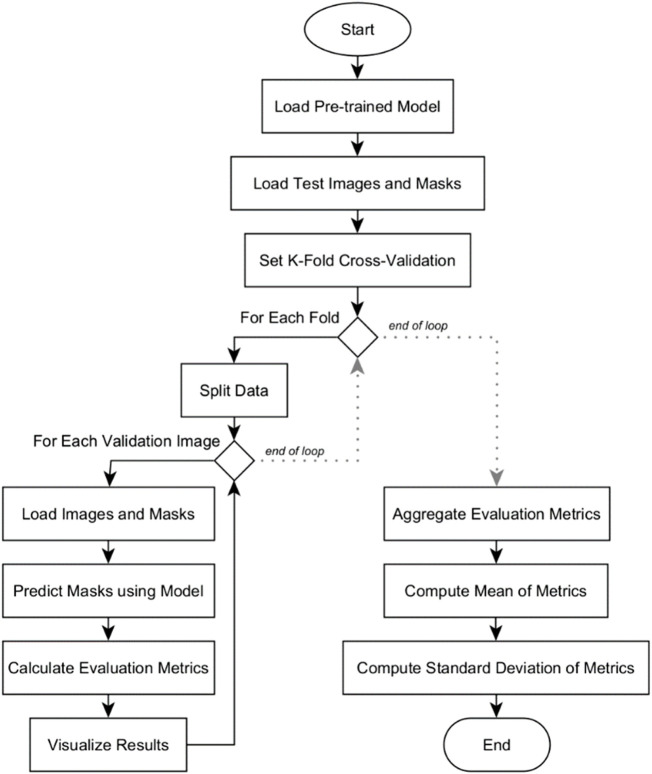
Flowchart of k-fold cross-validation analysis for model’s quantitative evaluation.

Quantitative analysis was conducted among the three deep learning models: U-Net, U-Net with Spatial Attention, and U-Net++. The flowchart in Figure 7 outlines the systematic process of conducting k-fold cross-validation analysis for evaluating the three pre-trained segmentation models.

The evaluation begins by loading the pre-trained model. Test images and their corresponding masks are then loaded, and the dataset is divided into subsets for k-fold cross-validation. Within each fold, the data is further split into training and validation sets. For each validation image, it is resized to match the model’s input shape and then preprocessed. The model predicts masks for these images, which are converted into binary format. Various evaluation metrics are calculated, and the results, including the original image, true mask, and predicted mask, are visualized for inspection. After evaluating all validation images in a fold, the metrics are aggregated. Mean and standard deviation of the metrics are computed across all folds. The results are presented in Section 5.2. These provide a comprehensive overview of the model’s overall performance and its consistency across different subsets of the dataset.

#### 3.5.1 Evaluation metrics

The following metrics are considered to evaluate the segmentation accuracy of the models, where:

TP (True Positives) are the pixels that are correctly classified as positive, 

FP (False Positives) are the pixels that are incorrectly classified as positive, 

TN (True Negatives) are the pixels that are correctly classified as negative, 

FN (False Negatives) are the pixels that are incorrectly classified as negative.

#### 3.5.2 Intersection over union (IoU)

This metric given in Equation 18 evaluates the overlap between the predicted and true masks. A higher IoU indicates better segmentation accuracy.

$$\text{IoU}=\frac{\text{TP}}{\text{TP}+\text{FP}+\text{FN}}$$ (18)

##### 3.5.2.1 Dice coefficient

The Dice coefficient given in Equation 19 is another measure of the overlap between two binary images, which provides insights into the model’s precision and sensitivity.

$$\text{Dice}=\frac{2\times \text{TP}}{2\times \text{TP}+\text{FP}+\text{FN}}$$ (19)

##### 3.5.2.2 Precision and recall

Precision quantifies the number of correct positive predictions made by the model, as given by Equation 20, while recall, in Equation 20, measures the model’s ability to identify all positive instances.

$$\text{Precision}=\frac{\text{TP}}{\text{TP}+\text{FP}}$$ (20)

$$\text{Recall}=\frac{\text{TP}}{\text{TP}+\text{FN}}$$ (21)

##### 3.5.2.3 Sensitivity and specificity

Sensitivity in Equation 22 gauges the model’s ability to correctly identify positive instances, whereas specificity in Equation 23 evaluates the model’s performance in correctly identifying negative instances.

$$\text{Sensitivity}=\frac{\text{TP}}{\text{TP}+\text{FN}}$$ (22)

$$\text{Specificity}=\frac{\text{TN}}{\text{TN}+\text{FP}}$$ (23)

##### 3.5.2.4 Pixel accuracy

This metric in Equation 24 determines the percentage of pixels that are correctly classified, offering a straightforward measure of the model’s accuracy at the pixel level.

$$\text{Pixel Accuracy}=\frac{\text{TP}+\text{TN}}{\text{TP}+\text{TN}+\text{FP}+\text{FN}}$$ (24)

##### 3.5.2.5 ROC-AUC

The Receiver Operating Characteristic Area Under the Curve provides a measure of the model’s ability to distinguish between the classes, with a value closer to 1 indicating superior performance.

##### 3.5.2.6 PR-AUC

The Precision-Recall Area Under the Curve evaluates the model’s precision-recall trade-off, especially useful when classes are imbalanced.

## 4 Qualitative analysis

**FIGURE 8:**
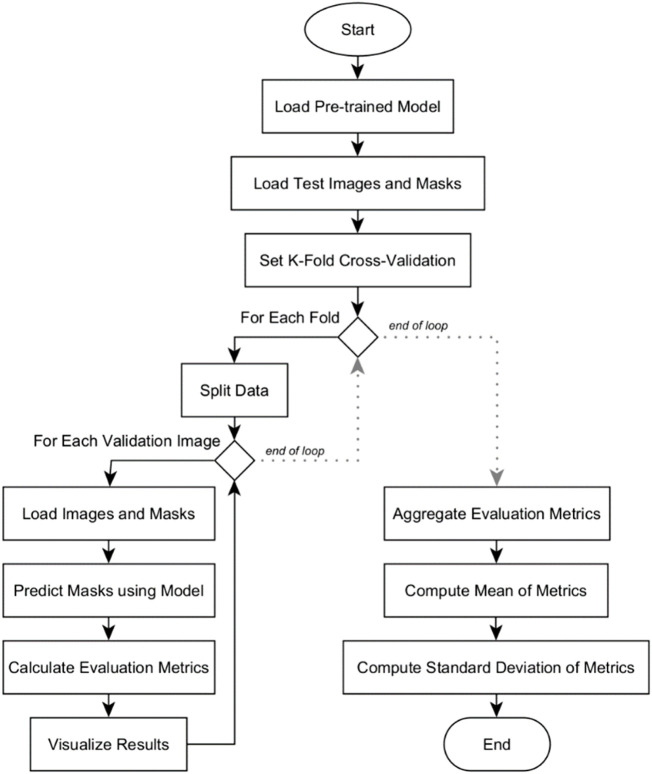
Flowchart of Grad-CAM heatmap visualization for model’s qualitative evaluation.

A qualitative analysis of the segmentation results generated by the three pre-trained segmentation models is performed through the utilization of Grad-CAM (Gradient-weighted Class Activation Mapping) heatmaps. The process of Grad-CAM heatmap visualization is outlined in the flowchart of Figure 8.

Grad-CAM heatmaps serve as a crucial tool for understanding the decision-making process of deep learning models, particularly in the context of image segmentation. The Grad-CAM heatmap visualization begins with the loading of the pre-trained segmentation model. Grayscale test images, representative of thermal data, are then loaded into the system. Each test image undergoes resizing to align with the pre-trained segmentation model’s input dimensions, accompanied by pre-processing steps to ensure compatibility with the model’s expectations. Subsequently, the pre-trained model processes the loaded test images, generating predictions while employing Grad-CAM to visualize regions of interest significantly contributing to the model’s decision.

The Grad-CAM heatmap computation involves leveraging gradients of the target class, specifically features indicative of breast tissue, with respect to the model’s final convolutional layer. These gradients are globally average-pooled to derive importance weights for each feature map. The identification of regions of interest is then accomplished by using these weights to highlight areas crucial for the model’s decision-making. The ensuing step involves overlaying the generated Grad-CAM heatmaps onto the original grayscale images, visually elucidating the correspondence between highlighted regions and actual features in the thermal images. This overlay process is systematically repeated for all test images, facilitating a comprehensive qualitative analysis of the model’s predictions and the corresponding regions of interest.

Grad-CAM heatmaps serve as a crucial tool for understanding the decision-making process of deep learning models, particularly in the context of image segmentation. The Grad-CAM heatmap visualization begins with the loading of the pre-trained segmentation model. Grayscale test images, representative of thermal data, are then loaded into the system. Each test image undergoes resizing to align with the pre-trained segmentation model’s input dimensions, accompanied by pre-processing steps to ensure compatibility with the model’s expectations. Subsequently, the pre-trained model processes the loaded test images, generating predictions while employing Grad-CAM to visualize regions of interest significantly contributing to the model’s decision. The Grad-CAM heatmap computation involves leveraging gradients of the target class, specifically features indicative of breast tissue, with respect to the model’s final convolutional layer. These gradients are globally average-pooled to derive importance weights for each feature map. The identification of regions of interest is then accomplished by using these weights to highlight areas crucial for the model’s decision-making. The ensuing step involves overlaying the generated Grad-CAM heatmaps onto the original grayscale images, visually elucidating the correspondence between highlighted regions and actual features in the thermal images. This overlay process is systematically repeated for all test images, facilitating a comprehensive qualitative analysis of the model’s predictions and the corresponding regions of interest.

Grad-CAM involves computations that are succinctly expressed through the following formulas (Selvaraju et al., 2016).

### 4.1 Gradient-weighted global average pooling

Grad-CAM calculates the importance weights by performing global average pooling on the gradients of the target class with respect to the feature maps. This is mathematically represented by Equation 25 as:

$$\alpha_{k}^{c}=\frac{1}{Z}\sum_{i}\sum_{j}\frac{\partial Y^{c}}{\partial A_{ij}^{k}}$$ (25)

Where:

$\alpha_{k}^{c}$ is the importance weight for the k− th feature map in the c− th class, 

Z is the normalization factor, 

$Y^{c}$ is the final prediction score for class c , 

$A_{ij}^{k}$ is the activation in the k− th feature map at position ( i,j ).

### 4.2 Weighted sum of feature maps

The weighted sum of feature maps is computed using Equation 26 to obtain the heatmap, denoted as $L_{\text{Grad}‐\text{CAM}}^{c}$ :

$$L_{\text{Grad}‐\text{CAM}}^{c}=\text{ReLU}\left(\sum_{k}\alpha_{k}^{c}A^{k}\right)$$ (26)

Where:

$A^{k}$ represents the k -th feature map.

### 4.3 Overlaying heatmap onto original image

The overlay operation in Equation 27 involves combining the Grad-CAM heatmap $\left(L_{\text{Grad}‐\text{CAM}}^{c}\right)$ with the original image $\left(I\right)$ :

$$\text{Resultant}\;\text{Image}=\text{Heatmap}\;\text{Weight}\times L_{\text{Grad}‐\text{CAM}}^{c}+\left(1-\text{Heatmap}\;\text{Weight}\right)\times I$$ (27)

Where:

The Heatmap Weight determines the intensity of the heatmap overlay.

The Grad-CAM heatmap visualization offers valuable insights into the interpretability of the model. It aids in understanding which regions of the input images are pivotal for the model’s predictions, thereby contributing to the overall assessment of the model’s performance in thermography-based breast region segmentation. The results of the qualitative analysis are presented in Section 5.3.

#### 4.3.1 Evaluation criteria

The segmentation outputs generated by each model are visually inspected from the Grad-CAM heatmaps to qualitatively assess the performance. The following criteria are analyzed:

##### 4.3.1.1 Breast region overlap

The extent to which the Grad-CAM heatmap aligns with the actual breast region in the thermal images is examined. The following scoring system is employed:

5 (Excellent): The Grad-CAM heatmap effectively highlights the component of breast region, aligning precisely with the breast boundaries.
4 (Good): The heatmap predominantly covers the breast area with minor inconsistencies in the highlighting.
3 (Moderate): The heatmap shows activations over parts of the breast, but with gaps or inaccuracies.
2 (Poor): Activations in the heatmap are sparse over the breast region, lacking coverage, and accuracy.
1 (Very Poor): The heatmap does not effectively highlight the breast region, lacking clear correlation with the actual boundaries.

##### 4.3.1.2 Noise Handling

The presence of noise or random activations in non-relevant areas of the Grad-CAM heatmap is observed:

5 (Excellent): There is minimal to no noise, with activations concentrated on the breast area.
4 (Good): There are a few minor instances of noise, limited and not significantly affecting the heatmap quality.
3 (Moderate): Some noise is present in non-relevant areas but does not obscure the breast region entirely.
2 (Poor): Noticeable noise patterns interfere with the clear depiction of the breast region.
1 (Very Poor): The heatmap is predominantly noisy with little meaningful activation in the breast region, making accurate identification impossible.

The color scheme used in the generated heatmaps utilized ‘jet’ colormap, where cool colors show low activations, and warm colors represent high activations. The interpretation of these colors is aligned with the model’s confidence levels, with warmer colors indicating higher confidence in the presence of breast tissue. The following aspects are considered:

1. Cool Colors (Blue/Green): Regions in the heatmap represented by cooler colors indicate low activations. These areas might correspond to regions where the model is less certain about the presence of breast tissue. The alignment of these low activation areas with non-breast regions or ambiguous features is examined.
2. Warm Colors (Yellow/Red): Areas in the heatmap represented by warmer colors indicate high activations. These regions correspond to the areas where the model is most confident about the presence of breast tissue. The accuracy of these high activation areas in capturing the actual breast tissue is assessed.
3. Transition Zones (Green to Yellow to Red): Transitional areas between cool and warm colors are analyzed. Smooth transitions from cool to warm colors along the boundaries of the breast tissue indicate gradual changes in activation levels, demonstrating accurate localization and segmentation.

## 5 Results

As stated, the annotations were carried out solely for experimental purposes and not for clinical application, since they were not performed by certified technicians. To mitigate potential bias, we followed standardized guidelines and performed cross-verification among the authors to ensure consistency and accuracy of the masks. We believe that this limitation does not compromise the reliability of the reported findings. Nevertheless, we fully agree that the inclusion of annotations from certified medical experts would add another layer of validation, and we consider this an important direction for future work.

### 5.1 Model training and evaluation results

**FIGURE 9:**
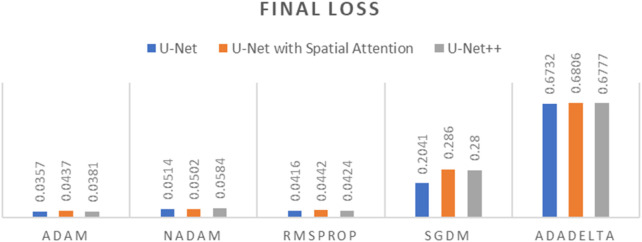
Graph of Final Loss of different optimizers across U-Net, U-Net with Spatial Attention, and U-Net++ Models.

**FIGURE 10:**
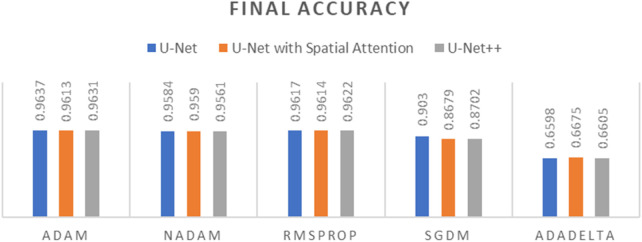
Graph of Final Accuracy of different optimizers across U-Net, U-Net with Spatial Attention, and U-Net++ Models.

**FIGURE 11:**
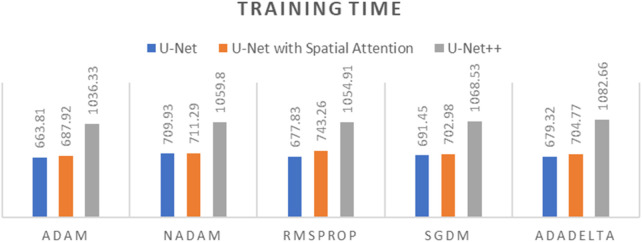
Graph of Training time of different optimizers across U-Net, U-Net with Spatial Attention, and U-Net++ Models.

To determine the optimal optimizer for training the deep learning models, a thorough comparative evaluation was conducted using five different optimizers: ADAM, NADAM, RMSPROP, SGDM, and ADADELTA. The evaluation focused on three key metrics: final loss, final accuracy, and training time. These metrics provide insight into the efficacy and efficiency of each optimizer in training the segmentation models. The final loss measures how well the model fits the training data, the final accuracy indicates the proportion of training data correctly classified by the model, and the training time reflects the optimizer’s computational efficiency. The results of this comparative evaluation are presented in Table 1–3. Which are also graphically represented in Figures 9–11.

**TABLE 1:** Final loss of different optimizers across U-net, U-net with spatial attention, and U-Net++ models.

Optimizer	U-Net	U-Net with spatial attention	U-Net++
ADAM	0.0357	0.0437	0.0381
NADAM	0.0514	0.0502	0.0584
RMSPROP	0.0416	0.0442	0.0424
SGDM	0.2041	0.2860	0.2800
ADADELTA	0.6732	0.6806	0.6777

**TABLE 2:** Final accuracy of different optimizers across U-net, U-net with spatial attention, and U-Net++ models.

Optimizer	U-Net	U-Net with spatial attention	U-Net++
ADAM	0.9637	0.9613	0.9631
NADAM	0.9584	0.9590	0.9561
RMSPROP	0.9617	0.9614	0.9622
SGDM	0.9030	0.8679	0.8702
ADADELTA	0.6598	0.6675	0.6605

**TABLE 3:** Training time of different optimizers across U-net, U-net with spatial attention, and U-Net++ models.

Optimizer	U-Net	U-Net with spatial attention	U-Net++
ADAM	663.81 s	687.92 s	1,036.33 s
NADAM	709.93 s	711.29 s	1,059.80 s
RMSPROP	677.83 s	743.26 s	1,054.91 s
SGDM	691.45 s	702.98 s	1,068.53 s
ADADELTA	679.32 s	704.77 s	1,082.66 s

The findings presented demonstrate significant variations in the efficacy of different optimization algorithms when applied to the three distinct deep learning models, U-Net, U-Net with Spatial Attention, and U-Net++. ADAM emerges as the preeminent optimizer for these models, consistently yielding the most favorable outcomes in terms of reduced loss values and heightened accuracy scores. The U-Net model, when trained using the ADAM optimizer, demonstrated notable performance with a final loss of 0.0357 and an accuracy of 0.9637. These results are highly competitive when compared to the others. In relation to the duration of the training, both the U-Net and U-Net with Spatial Attention architectures exhibit a notable level of efficiency, demonstrating comparable or reduced training times when compared to the U-Net++ model, regardless of the optimizer employed. The U-Net++ model exhibits a consistently longer training duration, which can be attributed to its intricate architectural design. The NADAM and RMSPROP optimizers exhibit comparable performance, albeit with marginally elevated loss values and diminished accuracy scores in comparison to the ADAM optimizer. In contrast, SGDM demonstrates notably elevated loss values and diminished accuracy scores across all three models, suggesting that it may not be the optimal selection for these specific models. Among the five optimizers, ADADELTA exhibits the poorest performance, characterized by significantly elevated loss values and notably decreased accuracy scores, along with relatively long training durations.

Using 20%–30% of the data for validation is a common practice in deep learning and medical image classification studies, as it provides a balance between training and validation sizes (Szegedy et al., 2016). While this proportion allows for an initial assessment of model performance, we acknowledge that the dataset size remains limited for testing the model’s generalizability to new or unseen data. Regarding the fixed 30 training epochs, this number was chosen to maintain consistency across all models and optimizers, with careful monitoring of training and validation loss to ensure convergence and prevent overfitting. Through our experiments, these settings proved suitable for all models to achieve optimal performance within the current dataset scope (Ding et al., 2022).

**FIGURE 12:**
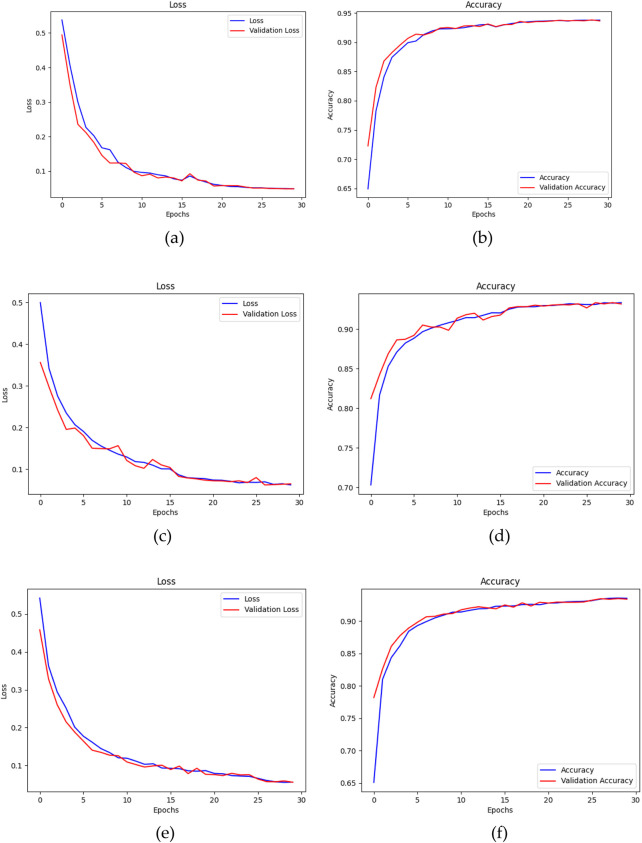
**(a)** Loss and **(b)** Accuracy of U-Net, **(c)** Loss and **(d)** Accuracy of U-Net with Spatial Attention, and **(e)** Loss and **(f)** Accuracy of U-Net++ over number of epochs using ADAM optimizer.

In conclusion, the ADAM optimizer is implemented for the training of the three segmentation models for its superior performance in this study. The training process of the models, using the ADAM optimizer, is visualized using their loss and accuracy graphs over the number of epochs. Figure 12, depict the convergence of each model throughout the training process. The training loss metric serves as a measure of the model’s ability to fit the data, whereas the accuracy metric reflects the frequency with which the model’s predictions align with the actual outcomes. Over the course of 30 epochs, the models demonstrated a progressive decline in the loss values and a steady improvement in accuracy, while guided by the ADAM optimizer.

### 5.2 Quantitative analysis results

K-fold cross-validation analysis was conducted to evaluate the three pre-trained segmentation models using 30% of the entire dataset. Detailed metrics, as outlined in Section 4.5, were meticulously examined. The evaluation results for U-Net, U-Net with Spatial Attention, and U-Net++ are presented in Tables 4–6, respectively. Each table provides a detailed breakdown of metrics such as Intersection over Union (IoU), Dice coefficient, precision, recall, sensitivity, specificity, pixel accuracy, ROC-AUC, and PR-AUC for every fold (k = 1–10).

**TABLE 4:** Evaluation metrics for the breast region segmentation folds using U-net model.

Metric	k = 1	k = 2	k = 3	k = 4	k = 5	k = 6	k = 7	k = 8	k = 9	k = 10
IoU	0.9283	0.9393	0.9421	0.9084	0.9135	0.9374	0.9396	0.9282	0.9464	0.9087
Dice Coefficient	0.9627	0.9687	0.9702	0.9513	0.9544	0.9676	0.9688	0.9627	0.9725	0.9514
Precision	0.9545	0.9862	0.9875	0.8681	0.9889	0.9867	0.9914	0.9903	0.9753	0.9922
Recall	0.9380	0.9619	0.9583	0.9652	0.9388	0.9549	0.9552	0.9568	0.9749	0.9549
Sensitivity	0.9380	0.9619	0.9583	0.9652	0.9388	0.9549	0.9552	0.9568	0.9749	0.9549
Specificity	0.9737	0.9876	0.9924	0.9087	0.9950	0.9908	0.9949	0.9926	0.9729	0.9927
Pixel Accuracy	0.9605	0.9752	0.9792	0.9304	0.9768	0.9758	0.9799	0.9768	0.9740	0.9741
ROC-AUC	0.9559	0.9747	0.9753	0.9369	0.9669	0.9728	0.9751	0.9747	0.9739	0.9738

**TABLE 5:** Evaluation metrics for the breast region segmentation folds using U-net with spatial attention model.

Metric	k = 1	k = 2	k = 3	k = 4	k = 5	k = 6	k = 7	k = 8	k = 9	k = 10
IoU	0.9267	0.9235	0.9422	0.9239	0.9239	0.9451	0.9373	0.9230	0.9316	0.9125
Dice Coefficient	0.9617	0.9601	0.9702	0.9601	0.9603	0.9717	0.9676	0.9598	0.9646	0.9535
Precision	0.9216	0.9782	0.9893	0.8966	0.9633	0.9849	0.9907	0.9764	0.9634	0.9924
Recall	0.9595	0.9665	0.9644	0.9769	0.9644	0.9704	0.9583	0.9641	0.9690	0.9593
Sensitivity	0.9595	0.9665	0.9644	0.9769	0.9644	0.9704	0.9583	0.9641	0.9690	0.9593
Specificity	0.9520	0.9801	0.9934	0.9299	0.9825	0.9893	0.9945	0.9816	0.9597	0.9928
Pixel Accuracy	0.9548	0.9736	0.9822	0.9479	0.9767	0.9814	0.9808	0.9739	0.9646	0.9763
ROC-AUC	0.9558	0.9733	0.9789	0.9534	0.9735	0.9799	0.9764	0.9728	0.9644	0.9761
PR-AUC	0.8992	0.9615	0.9679	0.8847	0.9405	0.9681	0.9652	0.9572	0.9498	0.9721

**TABLE 6:** Evaluation metrics for the breast region segmentation folds using U-Net++ model.

Metric	k = 1	k = 2	k = 3	k = 4	k = 5	k = 6	k = 7	k = 8	k = 9	k = 10
IoU*	0.9340	0.9352	0.9252	0.8994	0.9187	0.9469	0.9243	0.9113	0.9342	0.9215
Dice Coefficient	0.9657	0.9664	0.9610	0.9460	0.9575	0.9727	0.9606	0.9535	0.9659	0.9588
Precision	0.9294	0.9883	0.9732	0.8377	0.9749	0.9848	0.9877	0.9633	0.9707	0.9860
Recall	0.9616	0.9653	0.9690	0.9695	0.9489	0.9658	0.9575	0.9619	0.9651	0.9569
Sensitivity	0.9616	0.9653	0.9690	0.9695	0.9489	0.9658	0.9575	0.9619	0.9651	0.9569
Specificity	0.9571	0.9894	0.9832	0.8831	0.9884	0.9893	0.9927	0.9711	0.9680	0.9868
Pixel Accuracy	0.9588	0.9779	0.9777	0.9163	0.9756	0.9795	0.9794	0.9671	0.9665	0.9721
ROC-AUC*	0.9593	0.9774	0.9761	0.9263	0.9686	0.9775	0.9751	0.9665	0.9666	0.9719
PR-AUC*	0.9079	0.9706	0.9550	0.8239	0.9415	0.9654	0.9618	0.9434	0.9550	0.9648

The U-Net model exhibited robust performance with an average IoU of 0.9292 and a standard deviation of 0.0136. Notably, the Dice coefficient averaged at 0.9630, indicating a high degree of accuracy in segmentation. Precision, recall, and specificity consistently maintained their values across folds, underscoring the model’s effectiveness in classifying true positives and negatives. Pixel accuracy reached an average of 0.9703, signifying precise pixel-level segmentation. The model’s discriminative ability, as measured by ROC-AUC and PR-AUC, was substantial, averaging at 0.9680 and 0.9472, respectively.

The U-Net with spatial attention model exhibited competitive results, with an average IoU of 0.9290 and a low standard deviation of 0.0095. The Dice coefficient showed a mean value of 0.9630, underlining accurate segmentation. Precision and specificity demonstrated consistent values across folds, indicating reliable positive classification. The ROC-AUC and PR-AUC averaged 0.9704 and 0.9466, respectively, emphasizing the model’s strong discriminative ability.

The U-Net++ model showcased competitive performance, with an average IoU of 0.9251 and a standard deviation of 0.0128. The Dice coefficient reached an average of 0.9608, indicating accurate segmentation results. Precision and specificity displayed consistent values, highlighting the model’s ability to accurately classify positive samples. The ROC-AUC and PR-AUC, measuring the model’s discriminative ability, averaged 0.9665 and 0.9389, respectively.

**FIGURE 13:**
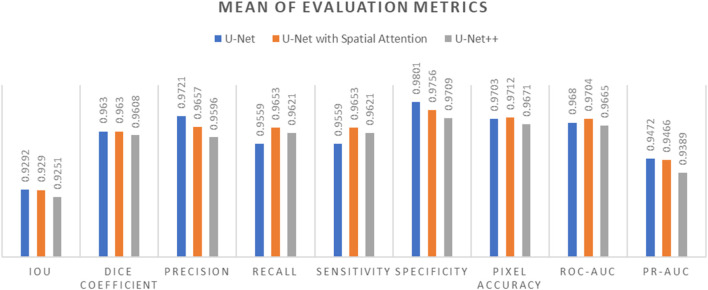
Mean of evaluation metrics chart for U-Net, U-Net with Spatial Attention, and U-Net++ models.

**FIGURE 14:**
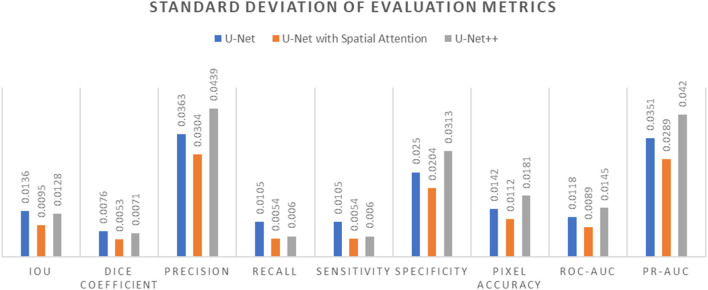
Standard Deviation of evaluation metrics chart for U-Net, U-Net with Spatial Attention, and U-Net++ models.

Comparative analysis of the results from each segmentation model is conducted by observing the mean and standard deviation for the evaluation metrics which are summarized in Tables 7 and 8, which are also graphically represented in Figures 13, 14.

**TABLE 7:** Mean of evaluation metrics for U-net, U-net with spatial attention, and U-Net++ models.

Metric	U-Net	U-Net with spatial attention	U-Net++
IoU	0.9292	0.9290	0.9251
Dice Coefficient	0.9630	0.9630	0.9608
Precision	0.9721	0.9657	0.9596
Recall	0.9559	0.9653	0.9621
Sensitivity	0.9559	0.9653	0.9621
Specificity	0.9801	0.9756	0.9709
Pixel Accuracy	0.9703	0.9712	0.9671
ROC-AUC	0.9680	0.9704	0.9665
PR-AUC	0.9472	0.9466	0.9389

**TABLE 8:** Standard deviation of evaluation metrics for U-net, U-net with spatial attention, and U-Net++ models.

Metric	U-Net	U-Net with spatial attention	U-Net++
IoU	0.9292	0.9290	0.9251
Dice Coefficient	0.9630	0.9630	0.9608
Precision	0.9721	0.9657	0.9596
Recall	0.9559	0.9653	0.9621
Sensitivity	0.9559	0.9653	0.9621
Specificity	0.9801	0.9756	0.9709
Pixel Accuracy	0.9703	0.9712	0.9671
ROC-AUC	0.9680	0.9704	0.9665
PR-AUC	0.9472	0.9466	0.9389

In terms of IoU, U-Net and U-Net with Spatial Attention demonstrate similarly high values of 0.9292 and 0.9290, respectively, with U-Net++ slightly lower at 0.9251. This metric reflects the degree of overlap between the predicted and ground truth segmentations, indicating the models’ effectiveness in capturing the target region.

The Dice Coefficient, another measure of segmentation accuracy, exhibits comparable performance among the models, with U-Net leading at 0.9630, followed closely by U-Net with Spatial Attention and U-Net++.

Precision, Recall, and Sensitivity metrics focus on different aspects of classification accuracy. U-Net consistently outperforms the other models in Precision, emphasizing its ability to minimize false positives. On the other hand, U-Net with Spatial Attention and U-Net++ show competitive performance in Recall and Sensitivity, highlighting their capacity to identify true positives.

Specificity measures the models’ ability to correctly identify true negatives, and U-Net maintains a slight advantage over the others in this regard. Pixel Accuracy, reflecting the overall accuracy of pixel-wise classification, indicates similar performance across the models.

The ROC-AUC and PR-AUC values, assessing the models’ discrimination and precision-recall trade-offs, exhibit minor variations among the models. Tables 4–6 provide the performance metrics measurement for UNet, UNet++ and UNet with spatial Attention and are discussed in Appendix A.

The standard deviations provided in Table 7 offer insights into the stability and consistency of each model’s performance across different metrics. Generally, U-Net demonstrates lower standard deviations compared to U-Net with Spatial Attention and U-Net++, suggesting more consistent results.

In summary, the evaluation metrics collectively suggest that U-Net performs competitively, demonstrating strong segmentation accuracy and consistency. U-Net with Spatial Attention and U-Net++ exhibit comparable performance, with slight variations in specific metrics. These findings contribute valuable information for selecting an appropriate model based on the desired trade-offs in thermography-based breast region segmentation.

### 5.3 Qualitative analysis results

**FIGURE 15:**
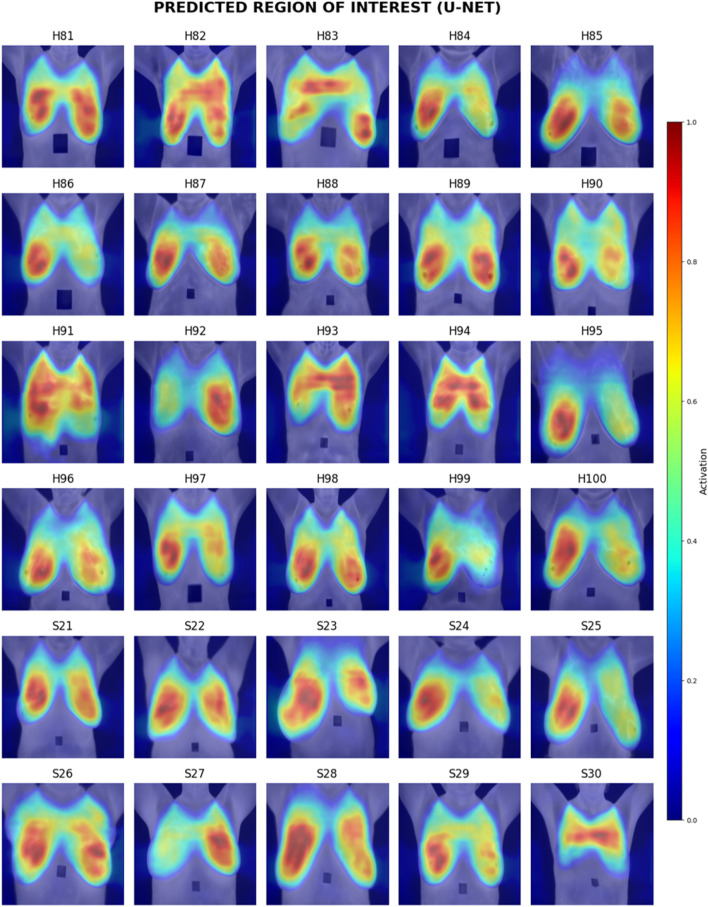
Grad-CAM heatmaps of Predicted Region of Interest for U-Net.

**FIGURE 16:**
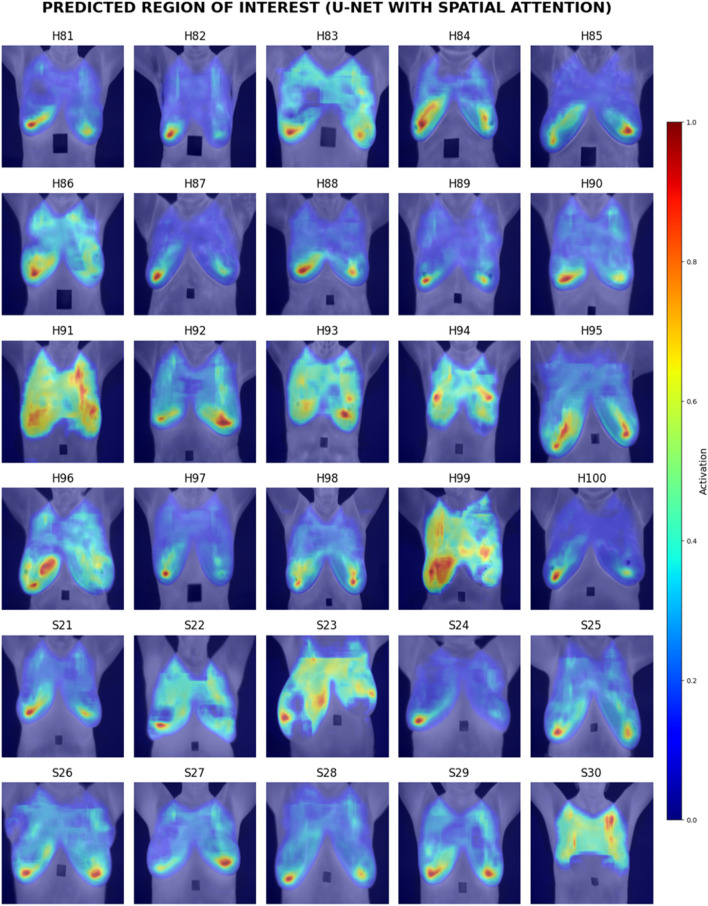
Grad-CAM heatmaps of Predicted Region of Interest for U-Net with Spatial Attention.

**FIGURE 17:**
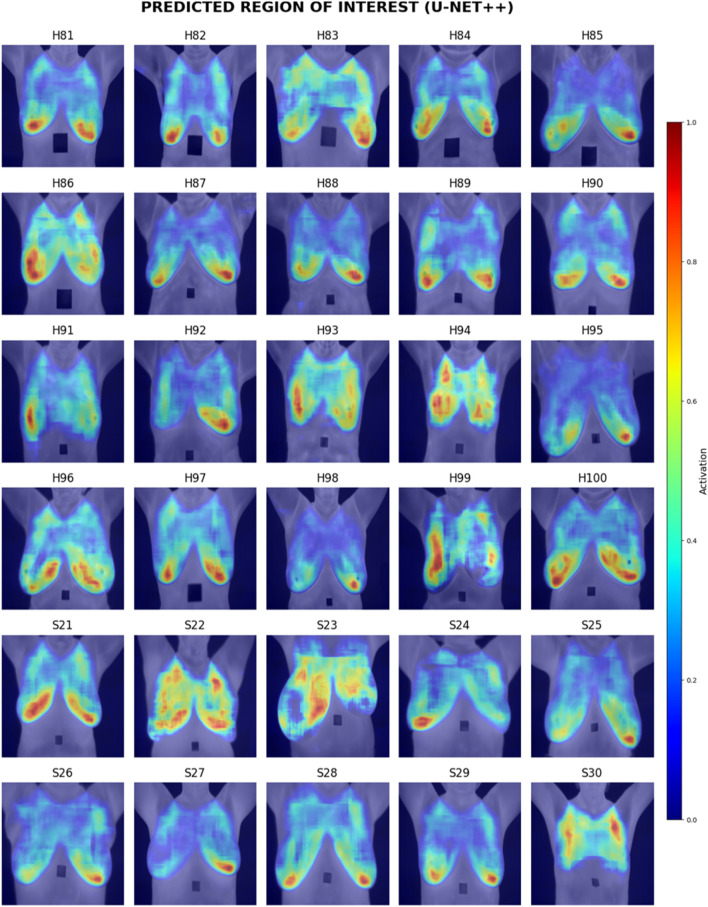
Grad-CAM heatmaps of Predicted Region of Interest for U-Net++.

Visual inspection of the segmentation results was conducted using the Grad-CAM heatmaps, focusing on the predicted region of interest generated by each model. Figures 15–17 display the Grad-CAM heatmaps for U-Net, U-Net with Spatial Attention, and U-Net++, respectively. The color patterns and transitions are observed from the heatmaps, providing a visual representation of how the model assigns importance to different areas in the thermal images. This visual inspection aids in understanding which regions the model identifies as crucial for predicting the presence of breast tissue, contributing to the interpretability of the model’s decision-making process.

Table 9 presents the comparative scores of Breast Region Overlap (BRO) and Noise Handling (NH) for U-Net, U-Net with Spatial Attention, and U-Net++ across 30% test images. The scores range from 1 (Poor) to 5 (Excellent) which were explained in Section 4.6.1.

**TABLE 9:** Comparative scores of breast region overlap (Bro) and noise handling (Nh) for U-net, U-net with spatial attention, and U-Net++ models across test images.

Test image	U-Net		U-Net with spatial attention		U-Net++	
	*BRO*	*NH*	*BRO*	*NH*	*BRO*	*NH*
H81	4	4	2	3	2	2
H82	5	5	2	2	2	2
H83	4	4	2	2	2	1
H84	5	5	2	3	2	2
H85	5	5	2	3	2	2
H86	4	5	3	2	2	2
H87	4	5	2	3	2	2
H88	4	5	2	3	2	2
H89	5	5	2	3	2	2
H90	4	4	2	2	2	2
H91	3	3	3	1	1	1
H92	3	3	2	2	2	2
H93	4	5	3	3	3	2
H94	3	5	2	3	3	2
H95	3	5	2	3	2	2
H96	4	5	2	2	3	2
H97	4	5	2	3	2	2
H98	4	5	2	3	2	1
H99	3	4	3	2	2	1
H100	4	5	2	3	2	2
S21	4	4	2	3	2	2
S22	5	5	2	2	3	2
S23	4	5	2	1	3	1
S24	4	5	2	3	2	2
S25	4	5	2	3	2	2
S26	5	5	1	2	2	2
S27	5	5	2	2	2	2
S28	5	5	2	3	2	2
S29	4	5	2	3	2	2
S30	3	5	2	3	2	2

**FIGURE 18:**
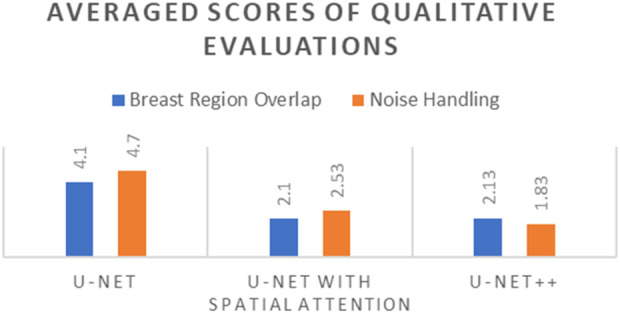
Averaged scores of qualitative evaluations for U-Net, U-Net with Spatial Attention, and U-Net++ Models.

Table 10 presents the comparative averaged scores of qualitative evaluations for the U-Net, U-Net with Spatial Attention, and U-Net++ models across two criteria: Breast Region Overlap and Noise Handling. A corresponding visual representation is provided in Figure 18, depicting the average scores for these models.

**TABLE 10:** Comparative averaged scores of qualitative evaluations for U-net, U-net with spatial attention, and U-Net++ models.

Criterion	U-Net	U-Net with spatial attention	U-Net++
Breast Region Overlap	4.10	2.10	2.13
Noise Handling	4.7	2.53	1.83

In terms of Breast Region Overlap, U-Net stands out with an impressive average score of 4.10, indicating a significant ability to align precisely with the breast boundaries in thermal images. On the other hand, both U-Net with Spatial Attention and U-Net++ exhibit lower average scores of 2.10 and 2.13, respectively, suggesting a diminished capability to accurately overlap with the actual breast region.

In terms of Noise Handling, U-Net excels with a high average score of 4.7, show-casing robust noise handling and concentrated activations on the breast area. In contrast, U-Net with Spatial Attention and U-Net++ struggle with noise, as indicated by their average scores of 2.53 and 1.83, respectively. These models face challenges in maintaining clarity in depicting the breast region due to noticeable noise patterns.

The overall analysis highlights U-Net’s superior performance in both Breast Region Overlap and Noise Handling compared to U-Net with Spatial Attention and U-Net++. Furthermore, U-Net with Spatial Attention and U-Net++ exhibit comparable performance, with U-Net++ showing a slight improvement.

## 6 Discussion

The results of the comprehensive evaluation of different optimizers for training deep learning models in breast region segmentation reveal notable variations in efficacy across U-Net, U-Net with Spatial Attention, and U-Net++. The ADAM optimizer consistently outperforms other algorithms, demonstrating reduced loss values and heightened accuracy scores. Surprisingly, the foundational U-Net, trained with ADAM, stands out in terms of effectiveness, challenging conventional assumptions regarding the necessity of architectural complexity for improved outcomes.

The competitive performance of U-Net, despite its foundational design, prompts a reconsideration of the presumed direct correlation between architectural complexity and segmentation accuracy. The nuanced perspective emerging from this study questions the prevailing notion that more intricate models necessarily yield superior results in the specific context of breast region segmentation in thermal images.

The comparative analysis of quantitative results across evaluation metrics provides valuable insights. U-Net exhibits strong segmentation accuracy and consistency, outperforming U-Net with Spatial Attention and U-Net++. Despite comparable outcomes in certain metrics, U-Net maintains lower standard deviations, indicating more stable and consistent performance.

The findings highlight the significance of U-Net’s foundational architecture, challenging assumptions about the need for complex models in breast region segmentation. The study’s outcomes contribute valuable information for selecting models based on desired trade-offs in thermography-based breast region segmentation.

Visual inspection of Grad-CAM heatmaps reinforces the study’s quantitative findings. U-Net’s impressive Breast Region Overlap and Noise Handling scores suggest its robustness in precisely aligning with breast boundaries and handling noise. In contrast, U-Net with Spatial Attention and U-Net++ face challenges in noise handling, indicating potential areas for improvement in these models.

The averaged scores further underscore U-Net’s superior performance in both criteria, highlighting its effectiveness in breast region segmentation. This aligns with the quantitative results and strengthens the argument for considering foundational U-Net as a viable option in this application.

The study opens avenues for future research by challenging established assumptions and providing a nuanced perspective on the relationship between model architecture, optimization strategies, and segmentation efficacy. Further investigations could explore the transferability of these findings to other medical imaging applications and datasets. Additionally, efforts to enhance the noise handling capabilities of more complex models like U-Net with Spatial Attention and U-Net++ may lead to improved overall performance.

In conclusion, this study challenges the *status quo* in deep learning for breast region segmentation by showcasing the effectiveness of the foundational U-Net with the ADAM optimizer. The findings have broader implications for the development of deep learning models in medical image analysis, encouraging researchers to reconsider the balance between model complexity and performance in specific applications. Table 11 compares performance of three models, showing that U-Net achieves highest boundary accuracy, robustness to noise, and faster training with greater stability when optimized with ADAM, making it the most effective for breast region segmentation. Although U-Net with Spatial Attention and U-Net++ offer marginal improvements in some quantitative metrics, they struggle more with noise handling and require longer, less stable training periods, with ADAM remaining the optimal optimizer across all models.

**TABLE 11:** Comparative chart summarizing the performance of U-Net, U-Net with Spatial Attention, and U-Net++.

Model	Key performance metrics	Qualitative observations	Observations on Noise handling	Training time and stability	Optimal optimizer
U-Net	-IoU (∼0.935–0.945) Dice (∼0.961–0.972) - Precision (∼0.929–0.987) - ROC-AUC ∼0.955–0.979	- Strong Boundary and Overlap scores - Robustness demonstrated via Grad-CAM heatmaps	- Handles noise effectively (scores ∼4.7/5 in qualitative assessment)	- (lower standard deviation) - Faster training (∼30 epochs)	ADAM
U-Net with Spatial Attention	- Slight improvements in some metrics but limited evidence of clear advantage	- Slightly better in some cases but faces challenges with noise	- Struggles with noise, noisier Grad-CAM heatmaps (∼2.53/5)	- Slightly longer training time; more complex; less stable	ADAM
U-Net++	- IoU (∼0.913–0.945) - Dice (∼0.953–0.971) - Precision (∼0.837–0.990)	- Slight improvement in some metrics but less transparent in noise handling	- Less effective noise suppression; higher noise artifacts observed	- Longer training durations due to architectural complexity	ADAM

The choice of optimizer, particularly ADAM, proved to be crucial across all models, with U-Net trained using ADAM consistently achieving the lowest loss (∼0.0357) and the highest average accuracy, demonstrating its effectiveness in minimizing errors and enhancing model performance. Grad-CAM heatmaps further highlighted that simpler models like U-Net more effectively delineate breast borders and exhibit greater resilience under noisy conditions, which is essential for medical imaging applications. Although attention mechanisms are generally intended to improve model focus on relevant regions, empirical results indicated they do not significantly outperform the baseline U-Net in noisy thermography images and may introduce additional training instability. Overall, this comparison suggests that the foundational U-Net—when optimized with ADAM—strikes an optimal balance of simplicity, robustness, interpretability, and computational efficiency, whereas the added architectural complexity of U-Net++ and attention-based models does not substantially enhance performance and may even create vulnerabilities in handling noisy thermal data for breast region segmentation.

### 6.1 Statistical validation and key insights

While the evaluation metrics demonstrate strong performance across all three U-Net variants, statistical validation is essential to assess whether the observed differences are significant. A pairwise Wilcoxon signed-rank test was applied across the folds of cross-validation for IoU and Dice scores, comparing U-Net against U-Net++ and U-Net with Spatial Attention. Results indicated no statistically significant improvement (p > 0.05) for the more complex models over baseline U-Net. This suggests that architectural sophistication does not guarantee superior outcomes in breast region segmentation using thermal images.

A critical insight from this study is the effectiveness of simpler models. The baseline U-Net with ADAM optimizer consistently produced high Dice (0.9630), IoU (0.9292), and specificity (0.9801) while maintaining computational efficiency and stability. These findings highlight that in medical image analysis, especially with limited datasets, robust optimization and careful training can outweigh added architectural complexity. Thus, for clinical or resource-constrained applications, standard U-Net trained with ADAM offers the best balance between accuracy, interpretability, and computational cost, making it a practical and reliable choice.

### 6.2 Novelty and contribution

This study makes a significant contribution to the field of thermography-based breast region segmentation by systematically evaluating and comparing the performance of three deep learning models—U-Net, U-Net with Spatial Attention, and U-Net++. The novelty of this research lies in its comprehensive analysis of the impact of different optimizers on model training, focusing on ADAM, NADAM, RMSPROP, SGDM, and ADADELTA. Beyond technical benchmarking, the study emphasizes dataset transparency, explicitly detailing the source, acquisition protocol, imaging device, and availability of the DMR-IR dataset, thereby ensuring reproducibility and reliability for future studies. A key finding is the superior performance of the baseline U-Net, particularly when trained with the ADAM optimizer. Despite being less complex than its variants, U-Net demonstrated high segmentation accuracy, interpretability through Grad-CAM, and reduced computational cost—highlighting that simplicity coupled with robust optimization can outperform architectural complexity.

From a clinical perspective, these results are highly relevant. U-Net’s ability to deliver strong precision and specificity reduces false positives, which is critical in breast cancer screening workflows. Meanwhile, the attention-based U-Net, with its improved sensitivity, may be suited to applications requiring the detection of subtle or ambiguous abnormalities. Together, these findings suggest that thermography, combined with deep learning segmentation, has potential as a low-cost adjunct to existing screening tools, particularly in resource-limited settings. This research contributes valuable insights into the selection of model architectures and optimizers for accurate and interpretable breast region segmentation in thermal images. The results provide a foundation for future research, guiding the development of advanced methodologies in medical imaging while also reinforcing the translational potential of thermography for clinical decision support. A key limitation of this study is that the manual annotations used to generate ground-truth masks were performed solely by the authors. Although cross-verification procedures were applied to minimize bias, the absence of certified radiologist annotations restricts the clinical validity of the segmentation masks. Future work will address this limitation by incorporating expert medical annotations to further strengthen reliability.

## Data Availability

Publicly available datasets were analyzed in this study. This data can be found here: http://visual.ic.uff.br/dmi.

## Appendix A

Since our results reported for each fold of the ten-fold cross-validation (Tables 4–6), we conducted a paired statistical analysis across the folds. We used the Friedman test for the three models, followed by pairwise Wilcoxon tests with Holm correction, and also reported effect sizes (Cohen’s dz). The analysis showed no statistically significant differences between the models in IoU and Dice across the folds (e.g., Friedman for IoU: p ≈ 0.90, Dice: p ≈ 0.84; all pairwise comparisons were non-significant after correction). This result is consistent with the small observed differences and the difficulty of achieving substantial improvement over the baseline U-Net model. We note that the tests were conducted on cross-validation folds, which are not fully independent, making the analysis conservative; hence, we used non-parametric paired tests to account for this.
